# The impact of natural selection on health and disease: uses of the population genetics approach in humans

**DOI:** 10.1111/eva.12045

**Published:** 2013-01-21

**Authors:** Estelle Vasseur, Lluis Quintana-Murci

**Affiliations:** 1 Institut Pasteur, Unit of Human Evolutionary Genetics75015, Paris, France; 2 Centre National de la Recherche Scientifique, URA 301275015, Paris, France; 3 Centre National de la Recherche Scientifique, UMR 5174, Evolution et Diversité Biologique31062, Toulouse, France; 4 Université de Toulouse31062, Toulouse, France

**Keywords:** evolution, genetic adaptation, human, natural selection, population genetics.

## Abstract

Investigations of the legacy of natural selection in the human genome have proved particularly informative, pinpointing functionally important regions that have participated in our genetic adaptation to the environment. Furthermore, genetic dissection of the intensity and type of selection acting on human genes can be used to predict involvement in different forms and severities of human diseases. We review here the progress made in population genetics studies toward understanding the effects of selection, in its different forms and intensities, on human genome diversity. We discuss some outstanding, robust examples of genes and biological functions subject to strong dietary, climatic and pathogen selection pressures. We also explore the possible relationship between cancer and natural selection, a topic that has been largely neglected because cancer is generally seen as a late-onset disease. Finally, we discuss how the present-day incidence of some diseases of modern societies may represent a by-product of past adaptation to other selective forces and changes in lifestyle. This perspective thus illustrates the value of adopting a population genetics approach in delineating the biological mechanisms that have played a major evolutionary role in the way humans have genetically adapted to different environments and lifestyles over time.

## Introduction

Our understanding of the patterns of human genome diversity has improved considerably over the last 10 years. The complete sequence of the human genome, which was published in 2001 (Lander et al. 2001; Venter et al. 2001), provided us with information about the location and genomic structure of genes, but did little to improve our understanding of the genetic diversity of the human genome at population level. A number of international consortia have since investigated the genetic differences both between individuals from the same population and between different populations from around the world. The International HapMap project (International HapMap Consortium 2005; Frazer et al. 2007; Altshuler et al. 2010), based on genotyping technologies, and, more recently, the 1000 genomes project (1000 Genomes Project Consortium 2010), based on next-generation sequencing, have made an enormous contribution to the identification and characterization of different types of variation in different populations worldwide. The HapMap project, for example, has cataloged both allele frequencies and levels of genetic association (assessed by measuring linkage disequilibrium, LD) across several populations, for 3.5 million single nucleotide polymorphisms (SNPs) (International HapMap Consortium 2005; Frazer et al. 2007; Altshuler et al. 2010). The 1000 Genomes project has described the location and allele frequencies of approximately 15 million SNPs, 1 million short insertions and deletions, and 20 000 structural variants. Interestingly, the data obtained suggest that each of us carries 250–300 loss-of-function variants of known genes, and that we are heterozygous for 50–100 variants known to be involved in genetic disorders (1000 Genomes Project Consortium 2010; Abecasis et al. 2012).

These data sets for human genetic variation in health have also been very useful for the interpretation and design of genome-wide association studies (GWAS) on many human diseases. By the end of 2012, more than 1500 GWAS had been published on more than 250 traits or diseases as varied as height, hair color, obesity, smoking behavior, schizophrenia and colorectal or bladder cancer (see, National Human Genome Research Institute catalog of published GWA studies: http://www.genome.gov/26525384). These GWAS have made a major contribution to our understanding of the genetic basis of human phenotypic variation in both health and disease, by identifying genes/variants associated with the variation of traits of interest, disease susceptibility/severity and differences in response to treatment.

## The evolutionary and population genetics approach

Various factors shape the diversity of the human genome at the population level and may contribute to phenotypic variation. Mutation and recombination create and reshuffle, respectively, diversity within the chromosomes. Other factors, such as demographic processes and the cultural behavior of human populations, then affect allelic frequencies within populations (Salem et al. 1996; Seielstad et al. 1998; Chaix et al. 2004, 2007; Wilder et al. 2004). Both genetic and archeological evidence have supported a common, recent origin of all humans in Africa, followed by range expansion and dispersal out-of-Africa (i.e., the Out-of-Africa hypothesis) (Lewin 1987; Quintana-Murci et al. 1999; Cavalli-Sforza and Feldman 2003; Macaulay et al. 2005; Mellars 2006; Fagundes et al. 2007; Laval et al. 2010). However, there is increasing evidence to suggest that such dispersals of modern humans were accompanied by some degree of admixture with local populations of ancient hominids. Indeed, recent estimates suggest that Neanderthals contributed 1–4% to modern Eurasian genomes, and Denisovans contributed 4–6% to modern Melanesian genomes (Green et al. 2010; Reich et al. 2010) Furthermore, adaptive introgression of archaic HLA haplotypes from Neanderthal and Denisovan genomes have been documented among some modern human populations (Abi-Rached et al. 2011), highlighting overall that the Out-of-Africa hypothesis oversimplifies a much more complex scenario.

The colonization of new geographic regions led to the exposure of human populations to different, changing environments, and these differences in climate, nutritional resources or pathogens acted as selective forces, to which human populations had to adapt if they were to survive (Sabeti et al. 2006; Nielsen et al. 2007; Novembre and Di Rienzo 2009). This brings us neatly to the concept of natural selection, as genetic variants increasing fitness would have been conserved in such conditions, thereby increasing in frequency, whereas deleterious variants would have been rapidly eliminated, contributing to the process of genetic adaptation.

Investigations of the legacy of natural selection events in the past within the human genome and of the ways in which these selective events have shaped current genetic diversity have proved particularly informative, pinpointing functionally important regions of the genome (Bamshad and Wooding 2003; Akey et al. 2004; Bustamante et al. 2005; Sabeti et al. 2006, 2007; Voight et al. 2006; Barreiro et al. 2008; Pickrell et al. 2009; Altshuler et al. 2010). Indeed, studies of whether and how selection has targeted particular genes in the human species as a whole, or in specific human populations, constitute a powerful approach for identifying genes that have played (and probably continue to play) an essential biological role in our survival, and distinguishing between such genes and those with a higher degree of redundancy (Sabeti et al. 2006; Quintana-Murci et al. 2007; Barreiro and Quintana-Murci 2010). Furthermore, population genetic dissection of the intensity and type of selection acting on human genes facilitates the identification of genes likely to be involved in rare, severe Mendelian diseases and makes it easier to distinguish between these genes and those most likely to be involved in complex susceptibility to disease. Overall, this approach increases our understanding of the ways in which past selection events have contributed to current differences in resistance/susceptibility to disease (Blekhman et al. 2008).

## Natural selection acts in different forms

Natural selection can take many different forms and act with different intensities. The most common type of natural selection is probably *purifying selection*, also known as *negative selection*, which affects most genes, to various extents (Bamshad and Wooding 2003; Bustamante et al. 2005; Nielsen et al. 2005). Purifying selection decreases the frequency of mutations that prove to be disadvantageous to carriers in a given environment, the magnitude of this decrease depending on the degree to which the mutation is deleterious. If the effects of the variant are highly deleterious to carriers, or even lethal, the variant is purged from the population by strong purifying selection. The frequent removal of deleterious variants can also result in the occasional removal of neutral linked variation (particularly in low-recombining regions), a phenomenon known as *background selection*. Genes subject to this type of selection generally display a major deficit of non-synonymous mutations. Such genes are thus likely to play an essential role in the host, with few, if any, amino-acid substitutions tolerated. In this case, the occurrence of new amino-acid variants may lead to severe Mendelian diseases. Purifying selection is weaker for mutations with effects that are only mildly deleterious. This allows such mutations to accumulate at the population level and to be maintained at a low frequency. In this case, an excess of low-frequency alleles is generally observed for the gene concerned, with no additional signature. Genes evolving under weaker evolutionary constraints are generally thought to be involved in less important, or more redundant, biological functions than those evolving under strong purifying selection.

In some cases, the occurrence of new genetic variants may be advantageous in a given environment, increasing the fitness of individuals, and the frequency of such variants may therefore rise due to *positive selection*. This process is also known as *directional* or *Darwinian selection* (Sabeti et al. 2006; Nielsen et al. 2007). When an advantageous mutation increases in frequency in the population as a result of positive selection, linked neutral variation will be dragged along with it—a process known as *genetic hitchhiking*. As a consequence, variation that is not associated with the selected allele is eliminated, resulting in a *selective sweep* that leads to an overall reduction of genetic diversity around the selected site. In such cases, several molecular signatures may be observed, including a skew in the distribution of allele frequencies toward an excess of both low-frequency alleles and high-frequency derived alleles (i.e., derived alleles with respect to the corresponding chimpanzee alleles), and a transitory increase in the strength of linkage disequilibrium associated with the selected allele(s).

Finally, several alleles may coexist at a given locus if they are advantageous individually or together, due to the effects of *balancing selection* (Charlesworth 2006; Hurst 2009). This is a general type of selective regime that favors the maintenance of diversity in a population. There are two main mechanisms by which balancing selection preserves polymorphism: *heterozygote advantage* and *frequency-dependent selection*.

Studies of the effects of selection at the interspecies level (e.g., comparing the human and chimpanzee lineages) can improve our understanding of the genetic mechanisms and traits underlying speciation. In turn, studies of the effects of selection on specific human populations are particularly useful for the identification of genes/variants responsible for the phenotypic diversity observed in human populations, in health (e.g., stature, skin color, hair development) and disease (e.g., differential susceptibility to infectious diseases or differences in response to treatment or vaccination).

## Neutrality tests for detecting the effects of selection

Each type of selection leaves a distinctive molecular signature (e.g., nucleotide diversity, allele frequency spectrum, haplotype length) in the genomic concerned. Such molecular signatures can be detected with various statistical tests that can be broadly subdivided into those that search for selection at the inter-species level (e.g., human versus chimpanzee) and those that focus on particular aspects of within-species data (e.g., within the human lineage or between human populations).

## Detection of selection between species

Inter-species neutrality tests make use of data concerning the divergence between closely related species, such as humans and chimpanzees, to detect relatively ancient selective events, such as those occurring at the time at which the two species considered diverged. In some cases, these tests also take into account data concerning polymorphism within species, making it possible to detect both older and more recent selective events. Inter-species tests include the traditional d*N/*d*S* test (Yang 1998), the McDonald-Kreitman (MK) test (McDonald and Kreitman 1991), and its extension MKPRF (Sawyer and Hartl 1992; Bustamante et al. 2005). The d*N/*d*S* test detects selection acting on protein-coding loci by comparing the ratio of non-synonymous (*d*_*N*_) to synonymous (*d*_*S*_) substitutions. Under neutrality, synonymous and non-synonymous substitutions should occur at a similar rate and we would therefore expect d*N/*d*S* = 1. However, the negative selection of non-synonymous variants would result in d*N/*d*S* < 1, whereas the positive selection of such variants would result in d*N/*d*S* > 1. In the MK test, which also considers polymorphic data, the two classes of mutations are assumed to be evolutionarily equivalent: patterns of polymorphism and divergence should therefore be the same for both classes of mutations. Non-independence between the counts of non-synonymous and synonymous polymorphisms and fixed differences (i.e., variants that reach fixation and are different between species) is assessed by Fisher's exact test. An excess of fixed differences for non-synonymous mutations (assumed to be subject to selection) with respect to synonymous variants is typically considered indicative of adaptive evolution. In contrast, an excess of polymorphic non-synonymous variants may result from weak negative selection or local, population-specific positive selection.

## Detection of selection within and between human populations

Intra-species neutrality tests focus on the level of polymorphism within a single species, such as humans, and therefore detect selection events that have occurred more recently. They can be subdivided into distinct groups, each focusing on different aspects of the genetic data. Allele frequency spectrum-based tests determine whether the frequency spectrum of mutations conforms to the expectations of the standard neutral model, and are extensively reviewed elsewhere (Kreitman 2000; Nielsen et al. 2005). Deviations from neutrality in the distribution of allele frequencies within populations can be measured with various tests, including Tajima's *D*, Fu and Li's D* and F* and Fay and Wu's *H* tests (Tajima 1989; Fu and Li 1993; Fay and Wu 2000). Negative values of Tajima's *D* and Fu and Li's D* and F* generally indicate an excess of rare alleles, consistent with the occurrence of negative or positive selection, whereas positive values of these statistics typically reflect an excess of alleles of intermediate frequency, due to balancing selection. Furthermore, Fay and Wu's *H* can be used to detected positive selection events (i.e., selective sweep), through the demonstration of an excess of high-frequency derived alleles of the targeted genes (or linked variation).

Other statistics, such as *F*_ST_ statistics, are based on the level of genetic differentiation between populations historically exposed to different selection pressures (Cavalli-Sforza 1966; Lewontin and Krakauer 1973; Excoffier et al. 1992; Weir and Hill 2002). For example, geographically restricted positive selection tends to increase the degree of differentiation between a specific human population and other human populations, resulting in an increase in *F*_ST_ value at the selected locus. Conversely, balancing selection, negative selection or species-wide directional selection may result in an *F*_ST_ value lower than expected under a hypothesis of neutrality (Cavalli-Sforza 1966; Bamshad and Wooding 2003).

Finally, another group of neutrality tests focus on more recent positive selection events (i.e., <30 000 years), by examining the patterns of haplotype homozygosity (haplotype length) associated with particular alleles. These tests include the long-range haplotype (*LRH*), integrated haplotype score (*iHS*), and LD decay (LDD) tests (Sabeti et al. 2002; Voight et al. 2006; Tang et al. 2007). These tests are all based on the comparison of the population frequency of a given mutation with the length of the haplotypes around it. Under neutral evolution, new alleles take a long time to reach high frequencies in the population, and haplotype lengths around these variants decrease substantially during this time, due to recombination. Thus, common alleles are typically old and associated with short haplotypes. In contrast, a variant subject to recent positive selection would be expected to have an unusually long haplotype for its population frequency, because the advantageous allele increases in frequency too rapidly for recombination to have a major effect on haplotype length.

## Pitfalls due to the mimicking effects of demography

Factors other than selection, such as particular demographic processes, may also account for deviations from the neutral model. Some of the tests for detecting selection described above, including those based on the allele frequency spectrum in particular, are sensitive to the confounding effects of demography on genetic diversity patterns. For example, an excess of rare alleles, giving negative values of Tajima's *D* and Fu & Li's D* and F* statistics, may actually result from a sudden population expansion rather than from the effects of positive selection (Ptak and Przeworski 2002; Nielsen et al. 2005). Similarly, positive values of these tests are indicative not only of balancing selection but also of the presence of strong population structure within the study-population (i.e., the study-population is indeed subdivided into different subpopulations), which may increase the proportion of alleles of intermediate frequency in the population (Kreitman 2000; Voight et al. 2005).

However, it is possible to overcome these problems by applying a basic principle of population genetics: demographic events affect the whole genome, whereas natural selection acts more locally and is restricted to particular genomic regions. Thus, when considering the impact of demographic factors on patterns of diversity, demographic models based on multiple, non-coding regions of the genome, taking into account realistic scenarios for the demographic history of human populations (e.g., population expansion, bottlenecks) can be incorporated into neutral expectations (Schaffner et al. 2005; Voight et al. 2005; Fagundes et al. 2007; Laval et al. 2010). Similarly, empirical procedures can be used to compare the value of a given statistic for the gene of interest (e.g., Tajmas's *D*, *F*_ST_, etc.) with background expectations for that statistic generated from genome-wide data, which should reflect neutrality. Thus, simulation-based or empirical procedures can be used to distinguish between the effects of demographic factors and those of natural selection events targeting specific genomic regions, providing evidence of past adaptation to new climates, foods or pathogens.

## Adaptive phenotypes in human populations

In the last few years, the accumulation of massive sets of genome-wide genetic variation data for diverse human populations, as in the HapMap and 1000 Genomes projects (International HapMap Consortium 2005; Frazer et al. 2007; Altshuler et al. 2010; 1000 Genomes Project Consortium 2010), has made it possible to test blindly for the occurrence of selection throughout the entire genome. Genome-wide scans for selection have provided long lists of genes putatively targeted by positive selection, together with information about the genomic regions and biological functions most likely to have played a role in our adaptation to the environment (reviewed by (Akey 2009)). So far, candidate-gene approaches have provided the most convincing evidence for the action of natural selection on particular genes, particularly when functional evidence is also available, despite the bias inherent to such approaches due to the need to make assumptions at the outset concerning the genes likely to be subject to selection. Furthermore, one needs to be cautious when linking the patterns of selection observed with putative phenotypes, particularly when functional analyses are performed in laboratory animals, where the genes concerned may have very different functions with respect to humans.

## Adaptation to diet

Many examples of the genetic adaptation of humans to diet have been described, including milk consumption, starch-rich diets, and bitter-taste perception (Bersaglieri et al. 2004; Wooding et al. 2004, 2006; Balaresque et al. 2007; Perry et al. 2007; Tishkoff et al. 2007; Patin and Quintana-Murci 2008). Adaptation to milk consumption, through lactase persistence, is probably one of the best-known examples of natural selection in humans. A high concentration of lactase ensures that lactose is digested effectively during the first few weeks of life. The lactase gene is then repressed, resulting in residual levels (5–10% those just after birth) of the enzyme in adults (Swallow 2003). However, some populations, particularly those that have traditionally practiced cattle husbandry, maintain the ability to digest milk into adulthood. This ‘lactase persistence’ trait is present at high frequency in European, Middle Eastern and pastoralist African populations (reaching frequencies of up to 90% in northern Europe) (Swallow 2003).

The genetic basis of the lactase persistence is now well known; it is inherited as a dominant Mendelian trait in Europeans, with a mutation (C/T-13910) in the promoter of the lactase gene (LCT), increasing the expression of this gene (Enattah et al. 2002). The lactase-persistence -13910T allele displays clear signatures of recent positive selection in Europeans (i.e., it lies on a long-range haplotype that largely exceeds its expected length under neutrality) (Bersaglieri et al. 2004; Nielsen et al. 2005), and there is a strong correlation between the geographic distribution of this allele and historical milk consumption (Beja-Pereira et al. 2003). Furthermore, the estimated age of expansion of this allele is between 5000 and 12 000 years ago (Beja-Pereira et al. 2003), suggesting that lactase persistence emerged in response to the cultural innovation of dairying associated with an agricultural lifestyle.

Different results have been found among East African pastoralist populations that also present the lactase-persistence trait, as these populations do not present the -13910T allele. Instead, a different mutation in the LCT promoter region (**−**14010C), which also increases LCT expression, has been shown to be positively selected over the last 7000 years among East African pastoralists (Tishkoff et al. 2007). Altogether, these results demonstrate that the cultural trait of milk consumption has conferred a strong selective advantage in terms of human survival in different parts of the world. The most obvious selective advantage provided by the lactase-persistence trait is the possibility for lactose-tolerant individuals to access a valuable food source in times that were characterized by food shortage. An alternative, but not mutually exclusive, hypothesis is that given that lactose-intolerant individuals present syndromes such as water loss diarrhea, individuals harboring the lactase persistence-associated alleles may have had a strong selective advantage for survival, particularly in the nutritionally poor and pathogen-rich conditions of the past. Today, lactose-intolerance can still be a disadvantageous trait, as illustrated by the considerable mortality observed in non-tolerant African children following consumption of milk products from alimentary aid.

## Adaptation to climate

Another example of genetic adaptation to changing environments is provided by the exposure of ancestral populations to colder climates and lower levels of incident sunlight after early migrations out-of-Africa (Coop et al. 2009). These changes in climatic conditions led to variation in the quantity, type, and distribution of melanin in the skin, resulting in various levels of skin pigmentation (Jablonski and Chaplin 2000, 2012). Darker skin was favored in regions of strong UV irradiation, such as the African continent, due to the obvious protection it provides against photodamage (e.g., sunburn, melanoma, and basal and squamous cell carcinomas) (Kollias et al. 1991). Furthermore, darker skins protect against UV-induced photolysis of folate, a metabolite essential for the normal development of the embryonic neural tube and spermatogenesis (Branda and Eaton 1978; Jablonski and Chaplin 2000). In turn, the evolution of lighter skin may reflect either a relaxation of functional constraints or a selective advantage for lighter skins, in regions of low UV radiation. There is increasing evidence supporting the selective hypothesis for lighter skin, as it allowed higher levels of vitamin D photosynthesis in regions with lower levels of UV irradiation (Jablonski and Chaplin 2000, 2012). In addition to these biological factors, sexual selection has been proposed as a factor further contributing to selection for skin color in the human population (Harpending 2002).

A number of genes involved in skin pigmentation have been identified, with effects at various stages of the pigmentation pathway (i.e., from melanogenesis to the production and maintenance of melanosomes and the switch between eumelanin and pheomelanin production) (Shriver et al. 2003; Lamason et al. 2005; Stokowski et al. 2007; Sulem et al. 2007; Han et al. 2008; McGowan et al. 2008). There is increasing evidence to suggest that many of these pigmentation genes, such as *ASIP*, *OCA2*, *SLC24A5, MATP,* or *TYR*, have been subject to positive selection across the globe or in specific human populations (Lamason et al. 2005; Izagirre et al. 2006; Soejima et al. 2006; Myles et al. 2007; Norton et al. 2007; Pickrell et al. 2009). For example, the *SLC24A5* gene, for which the A111G variant is associated with lighter skin color, presents strong signals of positive selection in Europeans, based on *F*_ST_ values and the haplotype homozygosity surrounding this variant (Lamason et al. 2005; Izagirre et al. 2006; Myles et al. 2007; Norton et al. 2007; Barreiro et al. 2008; Pickrell et al. 2009). In this case, positive selection may have increased the frequency of this mutation to maximize cutaneous vitamin D synthesis in areas with lower levels of UV irradiation, such as Europe (Norton et al. 2007).

Another interesting case of selection in response to environmental conditions is adaptation to altitude to avoid hypoxia, a physiological stress of the body due to the lower levels of oxygen at altitude. Various populations that have historically lived at high altitude, such as Andeans from the Andean Altiplano and Tibetans from the Himalayan plateau, have unique sets of physiological and morphological characteristics (e.g., increased respiratory and heart rate, changes in pulmonary artery pressure, enlarged thorax) that have allowed them to adapt to high-altitude conditions, where oxygen concentrations are about 40% lower than those of sea level. The genetic basis of such adaptations to extreme environmental conditions has started to be deciphered only recently. Recent studies have provided evidence of positive selection targeting various genes or gene regions involved in oxygen metabolism and sensing in Andeans and Tibetans, the strongest signals of selection being observed in the *EPAS1*, *EGLN1*, *PRKAA1,* and *NOS2A* genes (Bigham et al. 2010; Yi et al. 2010). These studies have helped to delineate a number of functionally important loci responsible for the genetic adaptation to high altitude.

## Adaptation to pathogen pressures

Another important selective pressure that has confronted humans over time is that imposed by pathogens and infectious diseases. Indeed, pathogens have been, and still are in regions in which antibiotic treatment, vaccine administration and hygiene improvements are limited, a major cause of human mortality, thus exerting strong selective pressure on the human genome (Casanova and Abel 2005; Quintana-Murci et al. 2007; Barreiro and Quintana-Murci 2010; Sironi and Clerici 2010). Pathogen-driven balancing selection has been clearly demonstrated for the human leukocyte antigen (HLA) gene, the tremendous diversity of which is strongly correlated with residence in an area in which there are large numbers of pathogen species (Prugnolle et al. 2005).

Several studies have identified multiple signatures of selection in response to the presence of *Plasmodium* parasites, the agent responsible for malaria (Kwiatkowski 2005). For example, the high frequency of some hemoglobinopathies has been correlated with greater resistance to *Plasmodium falciparum* malaria: the HbS allele or ‘sickle cell trait’ is a textbook example of positive selection due to an infectious disease, increasing resistance to life-threatening forms of malaria by an order of magnitude when present in the heterozygous state (Allison 1954; Ackerman et al. 2005; Kwiatkowski 2005). Another example is provided by glucose-6-phosphate dehydrogenase (G6PD) deficiency. Patients with this condition have abnormally low levels of G6PD, which is particularly important for red blood cell metabolism. More than a hundred variants can lead to this deficiency, some of which are selected because they confer greater protection against *falciparum* or *vivax* malaria (Tishkoff et al. 2001; Saunders et al. 2002; Louicharoen et al. 2009). An extreme example is provided by the *DARC* gene, null alleles of which result in an absence of protein, preventing *Plasmodium vivax* from penetrating into host cells (Tournamille et al. 1995). Positive selection for the null allele has been demonstrated in sub-Saharan Africans, and this allele is almost fixed in some Central African populations, whereas it is virtually absent from populations originating from other parts of the world (Livingstone 1984; Hamblin and Di Rienzo 2000; Hamblin et al. 2002). *Plasmodium vivax*, or another pathogen using the same mode of entry into host cells, has been identified as the most probable source of selective pressure in this case (Hamblin and Di Rienzo 2000; Hamblin et al. 2002). The selective pressures imposed by malaria can also act at the micro-geographic scale and be variable among closely related human populations. The case of the Fulani from West Africa, who present a specific resistance to malaria with respect to other ethnic groups living in the same area, is a prime example (Modiano et al. 1999).

## Multiple mutations underlying similar adaptive phenotypes

It is interesting to note that many of the best-studied cases of genetic adaptation in humans have revealed multiple, independent mutations that confer an advantage to the same or similar selective pressures. Again, the lactase persistence trait not only constitutes one of the best documented cases of positive selection in the human genome but also it represents a clear example of convergent adaptation in response to a strong selective force related to diet. Indeed, different lactase-persistence alleles are found in Europe, the Middle East, and Africa, where they have increased in frequency independently, as a result of strong positive selection (Bersaglieri et al. 2004; Tishkoff et al. 2007; Enattah et al. 2008; Itan et al. 2010). Even within a given population (e.g., East African pastoralists), multiple mutations have been found to explain the lactase-persistence trait. Furthermore, the estimated times at which these mutations began to increase in frequency differ slightly between Europe and Africa (Bersaglieri et al. 2004; Tishkoff et al. 2007), and coincide with the cultural practice of pastoralism (and therefore milk consumption) in these populations. A possible explanation for this fascinating example of convergent adaptation within our species is that mutation rates to adaptive alleles might be high enough to allow novel adaptive mutations to arise in distinct geographic regions before any single variant spreads globally (Coop et al. 2009).

Other cases of multiple mutations in a given gene, or even mutations located in different genes, underlying similar adaptive phenotypes in humans include resistance to malaria, adaptation to high altitude, or skin pigmentation. Indeed, various G6PD-deficiency alleles have been independently targeted by positive selection in African, Mediterranean, or South-East Asian populations, as they conferred higher resistance to *falciparum* or *vivax* malaria (Tishkoff et al. 2001; Saunders et al. 2002; Louicharoen et al. 2009). Likewise, different genes have been found to explain adaptation to high altitude in Tibetans (*EPAS1*) or Andeans (*PRKAA1*, *NOS2A*), and for the only gene targeted by positive selection in both populations, *EGLN1*, the selected haplotypes markedly differ between the two populations (Bigham et al. 2010; Yi et al. 2010). This clearly suggests that Tibetans and Andeans have followed different evolutionary trajectories to adapt to the same pressure, high altitude. Finally, the case of light skin in Europeans and East Asians provides with another example of convergent evolution (Norton et al. 2007). Indeed, different genes, associated with lighter skin color in these populations, have been independently targeted by positive selection in Europeans (*SLC24A5, MATP*, and *TYR*) and East Asians (*ADAM17* and *ATRN*) (Lamason et al. 2005; Izagirre et al. 2006; Soejima et al. 2006; Myles et al. 2007; Norton et al. 2007; Pickrell et al. 2009). Although the role of sexual selection cannot be ruled out, these data support independent genetic mechanisms for evolution of skin color.

## The special case of cancer selection

The relationship between selection and cancer remains blurry (Leroi et al. 2003). Some cancers affect mostly children, such as acute lymphoblastic leukemia (Ribera and Oriol 2009), and so they may select for anti-cancer adaptations that reduce the chance of death. Furthermore, although most cancers occur after the reproductive age, the variance is large, extended to much younger ages corresponding to a non-negligible fraction of individuals (Couch and Weber 1996). With this in mind, the possibility that cancers exert a direct selection pressure cannot be ruled out. In this context, theoretical considerations predict that the risk of developing cancer for large and long-lived species should be higher, in comparison with short-lived organisms, but this does not seem to be the case (Caulin and Maley 2011). This observation, known as the Peto's paradox (Peto et al. 1975), has been used to suggest that large animals might have developed some mechanisms to resist to cancer (Leroi et al. 2003; Nagy et al. 2007; Caulin and Maley 2011), supporting the notion that cancer may have been indeed evolutionary counter-selected.

The long-held view that cancers do not generally affect individuals of reproductive age has constituted a real obstacle to the use of the evolutionary genetics approach in this field. As a consequence, only a few studies have addressed directly the question of how selection has affected the evolution of cancer-related genes. Among them, the case of *BRCA1* is worth mentioning, as this gene displays one of the strongest, replicated associations with breast and ovarian cancers (for a review, see (Lee and Boyer 2001)). Interestingly, *BRCA1* has been found to evolve under purifying selection in humans (Pavard and Metcalf 2007). With respect to the related *BRCA2* gene, which is strongly associated with the same diseases, it also appears to be evolving under purifying selection (Bustamante et al. 2005). These observations are particularly interesting, as hereditary forms of breast cancer have a high impact in young adults (Hall et al. 1990). We might therefore expect genes associated with cancers with a strong impact on young adults or children to be highly constrained by purifying selection.

Signatures of positive selection have also been reported for certain genes known to play a direct or indirect role in cancer risk. Some of these studies focused on signatures of selection between species, to highlight adaptive selection in humans, as a whole, with respect to other species. For example, at the inter-species level, *BRCA1* appears to evolve adaptively, with some specific codons in its DNA repair domain being targeted by positive selection (Huttley et al. 2000; Fleming et al. 2003; Pavlicek et al. 2004). This finding and the above-mentioned signature of purifying selection in humans (Pavard and Metcalf 2007) are not incompatible but instead they suggest that *BRCA1* evolved adaptively before human speciation (accumulating various amino-acid changes), subsequently becoming strongly constrained within the human lineage.

At the intra-species level, some studies, which have made use of polymorphic data in humans, have found signatures of positive selection at some genes. For example, a genomic scan of 142 genes for signals of recent positive selection identified the *PPP2R5E* gene, which is involved in the negative regulation of cell growth and division (Voight et al. 2006; Grochola et al. 2009). Another selection scan of 132 genes found strong signatures of positive or balancing selection in a region extending over more than 115 kb in European-Americans, the strongest signals being centered on *TRPV6* (Akey et al. 2004; Stajich and Hahn 2005). This gene has been shown to be up-regulated in prostate cancer and associated to an aggressive form of the disease (Wissenbach et al. 2001; Paiss et al. 2003). One of the most interesting examples is probably that of *UGT2B4*, which has been strongly associated with increased risk of breast cancer in Nigerians and, to a lesser extent, African Americans, and signatures of selection (balancing selection or recent positive selection) have been found in the upstream portion of the gene (Sun et al. 2011). Altogether, these examples suggest that there might be a direct or indirect relationship between cancer and selection, but the overall genome-wide proportion of cancer-related genes actually targeted by selection remains to be determined.

## Maladaptation as a consequence of past selection

There is increasing evidence to suggest that some diseases of modern societies, such as obesity, hypertension, inflammatory or autoimmune diseases, allergies or even cancers, may simply be a by-product of past adaptation to other selective forces and changes in lifestyle. Genetic modifications occur more slowly than changes in lifestyle. It has therefore long been suggested that ancient selection events may have favoured variants that are no longer advantageous and may even have become detrimental in modern societies.

## Changes in nutritional and pathogenic pressures over time

The ‘thrifty gene hypothesis’ was first introduced by James Neel (Neel 1962), who suggested that genes conferring a predisposition to diabetes (called ‘thrifty genes’) were historically advantageous but had become detrimental in the modern world. Some variants had been positively selected in the past because they favored the accumulation of larger amounts of fat, greatly increasing the likelihood of survival between famines. However, the change to a sedentary lifestyle and the increase in food abundance has increased the risk of developing type II diabetes in individuals carrying these variants today. Several studies have revealed particularly high risks of diabetes and high levels of obesity in populations that have recently rapidly switched to a ‘Western lifestyle’, such as the Native Americans of the United States (Joffe and Zimmet 1998).

Another example of maladaptation may reflect changes in the selective pressures imposed by infectious diseases over time (Barreiro and Quintana-Murci 2010; Sironi and Clerici 2010). Pathogens are still present, but improvements in hygiene and the use of antibiotics and vaccines have greatly weakened the selection pressures they impose, particularly in Western countries. A strong, exacerbated immune response, which may have been the best way to survive in pathogen-rich environments in the past, seems to have become a burden in modern societies, as an overly vigorous response increases the risk of developing inflammatory and autoimmune diseases (Le Souef et al. 2000; Sironi and Clerici 2010) Indeed, populations with a long-term tropical ancestry introduced into temperate regions appear to have a higher risk of developing allergic inflammatory diseases and asthma, as shown for African Americans and for Asians living in the United Kingdom (Gillam et al. 1989; Gold et al. 1993; Von Behren et al. 1999). In all these cases, ethnic background was found to have a greater impact than environment on differences in the prevalence of asthma (Gilthorpe et al. 1998; Miller 2000). From a population genetics standpoint, some alleles conferring a higher risk of inflammatory and autoimmune diseases have been shown to have been under strong selective pressure in the past (Barreiro and Quintana-Murci 2010). This observation supports the notion that the higher risk of developing inflammatory and autoimmune disorders may be a by-product of past selection in response to infectious disease (Sironi and Clerici 2010).

## Compromises during life

Another compromise that has been suggested for adaptation is antagonistic pleiotropy. Pleiotropy is defined as the situation in which one gene controls more than one phenotypic trait in an organism; ‘antagonistic pleiotropy’ is the term used when at least one trait is beneficial and another is detrimental. The antagonistic pleiotropy hypothesis was firstly proposed by George C. Williams in 1957 to explain senescence (Williams 1957). Indeed, variants that were advantageous early in life could become detrimental later on (Stearns et al. 2010), as genetic variation may affect several pathways at different ages. For example, high levels of testosterone in the bloodstream are associated with a greater fitness in early life, but are associated with a higher risk of developing prostate cancer at later stages (Gann et al. 1996). Another interesting case is birth weight: it has been suggested that selection for high birth weight, which is beneficial for survival early in life, is counterbalanced by a higher risk of various cancers later in life (Thomas et al. 2012).

## Conclusion

Population genetic studies have greatly improved our knowledge of the way in which humans have genetically adapted to variation in environmental pressures and changes in lifestyle over time. Likewise, the investigation of how natural selection has driven the evolution of particular genes and biological functions has proven a useful tool to inform the relationship between genetic diversity, adaptive phenotypes and disease. In this context, detailed studies combining population genetic approaches making use of the massive whole-genome sequence-based data sets for various human populations, such as the 1000 Genomes Project (1000 Genomes Project Consortium 2010; Abecasis et al. 2012), and genome-wide association studies of multiple diseases and other traits of interest are now required. In particular, much more work is needed with respect to the increasingly recognized role of copy number variation in human adaptation and disease phenotypes, as rapid gains and losses of genomic segments can have a substantial impact on phenotypic variation (Feuk et al. 2006; Girirajan et al. 2011; Iskow et al. 2012). Similarly, we still have much to learn from the integration of data from population genetics, epigenetics and epidemiological genetics studies in populations with different lifestyles and modes of subsistence (e.g., agriculture, hunter-gathering, sedentary, nomadic) or living in different environments (e.g., urban, rural, forest). Such multidisciplinary efforts are clearly required to clarify the relationship between natural selection and disease and to improve our understanding of the evolutionary mechanisms accounting for the present-day differences in disease susceptibility, resistance or progression observed. Ultimately, these integrative approaches are likely to be essential for dissection of the contribution of genotypic, epigenetic, and environmental variables to the current risk of many diseases, facilitating improvements in their diagnosis, prevention and treatment.

## References

[b1] Abecasis GR, Auton A, Brooks LD, DePristo MA, Durbin RM, Handsaker RE, Kang HM (2012). An integrated map of genetic variation from 1,092 human genomes. Nature.

[b2] Abi-Rached L, Jobin MJ, Kulkarni S, McWhinnie A, Dalva K, Gragert L, Babrzadeh F (2011). The shaping of modern human immune systems by multiregional admixture with archaic humans. Science.

[b3] Ackerman H, Usen S, Jallow M, Sisay-Joof F, Pinder M, Kwiatkowski DP (2005). A comparison of case-control and family-based association methods: the example of sickle-cell and malaria. Annals of Human Genetics.

[b4] Akey JM (2009). Constructing genomic maps of positive selection in humans: where do we go from here?. Genome Research.

[b5] Akey JM, Eberle MA, Rieder MJ, Carlson CS, Shriver MD, Nickerson DA, Kruglyak L (2004). Population history and natural selection shape patterns of genetic variation in 132 genes. PLoS Biologyogy.

[b6] Allison AC (1954). Protection afforded by sickle-cell trait against subtertian malareal infection. British Medical Journal.

[b7] Altshuler DM, Gibbs RA, Peltonen L, Dermitzakis E, Schaffner SF, Yu F, Bonnen PE (2010). Integrating common and rare genetic variation in diverse human populations. Nature.

[b8] Balaresque PL, Ballereau SJ, Jobling MA (2007). Challenges in human genetic diversity: demographic history and adaptation. Human Molecular Genetics.

[b9] Bamshad M, Wooding SP (2003). Signatures of natural selection in the human genome. Nature Reviews Genetics.

[b10] Barreiro LB, Quintana-Murci L (2010). From evolutionary genetics to human immunology: how selection shapes host defence genes. Nature Reviews Genetics.

[b11] Barreiro LB, Laval G, Quach H, Patin E, Quintana-Murci L (2008). Natural selection has driven population differentiation in modern humans. Nature Genetics.

[b12] Beja-Pereira A, Luikart G, England PR, Bradley DG, Jann OC, Bertorelle G, Chamberlain AT (2003). Gene-culture coevolution between cattle milk protein genes and human lactase genes. Nature Genetics.

[b13] Bersaglieri T, Sabeti PC, Patterson N, Vanderploeg T, Schaffner SF, Drake JA, Rhodes M (2004). Genetic signatures of strong recent positive selection at the lactase gene. American Journal of Human Genetics.

[b14] Bigham A, Bauchet M, Pinto D, Mao X, Akey JM, Mei R, Scherer SW (2010). Identifying signatures of natural selection in Tibetan and Andean populations using dense genome scan data. PLoS Genetics.

[b15] Blekhman R, Man O, Herrmann L, Boyko AR, Indap A, Kosiol C, Bustamante CD (2008). Natural selection on genes that underlie human disease susceptibility. Current Biology: CB.

[b16] Branda RF, Eaton JW (1978). Skin color and nutrient photolysis: an evolutionary hypothesis. Science.

[b17] Bustamante CD, Fledel-Alon A, Williamson S, Nielsen R, Hubisz MT, Glanowski S, Tanenbaum DM (2005). Natural selection on protein-coding genes in the human genome. Nature.

[b18] Casanova JL, Abel L (2005). Inborn errors of immunity to infection: the rule rather than the exception. Journal of Experimental Medicine.

[b19] Caulin AF, Maley CC (2011). Peto's Paradox: evolution's prescription for cancer prevention. Trends in Ecology & Evolution.

[b20] Cavalli-Sforza LL (1966). Population structure and human evolution. Proceedings of the Royal Society of London. Series B: Biological Sciences.

[b21] Cavalli-Sforza LL, Feldman MW (2003). The application of molecular genetic approaches to the study of human evolution. Nature Genetics.

[b22] Chaix R, Austerlitz F, Khegay T, Jacquesson S, Hammer MF, Heyer E, Quintana-Murci L (2004). The genetic or mythical ancestry of descent groups: lessons from the Y chromosome. American Journal of Human Genetics.

[b23] Chaix R, Quintana-Murci L, Hegay T, Hammer MF, Mobasher Z, Austerlitz F, Heyer E (2007). From social to genetic structures in central Asia. Current Biology.

[b24] Charlesworth D (2006). Balancing selection and its effects on sequences in nearby genome regions. PLoS Genetics.

[b26] Coop G, Pickrell JK, Novembre J, Kudaravalli S, Li J, Absher D, Myers RM (2009). The role of geography in human adaptation. PLoS Genetics.

[b27] Couch FJ, Weber BL (1996). Mutations and polymorphisms in the familial early-onset breast cancer (BRCA1) gene. Breast Cancer Information Core. Human Mutation.

[b29] Enattah NS, Sahi T, Savilahti E, Terwilliger JD, Peltonen L, Jarvela I (2002). Identification of a variant associated with adult-type hypolactasia. Nature Genetics.

[b30] Enattah NS, Jensen TG, Nielsen M, Lewinski R, Kuokkanen M, Rasinpera H, El-Shanti H (2008). Independent introduction of two lactase-persistence alleles into human populations reflects different history of adaptation to milk culture. American Journal of Human Genetics.

[b31] Excoffier L, Smouse PE, Quattro JM (1992). Analysis of molecular variance inferred from metric distances among DNA haplotypes: application to human mitochondrial DNA restriction data. Genetics.

[b32] Fagundes NJ, Ray N, Beaumont M, Neuenschwander S, Salzano FM, Bonatto SL, Excoffier L (2007). Statistical evaluation of alternative models of human evolution. Proceedings of the National Academy of Sciences of the United States of America.

[b33] Fay JC, Wu CI (2000). Hitchhiking under positive Darwinian selection. Genetics.

[b34] Feuk L, Carson AR, Scherer SW (2006). Structural variation in the human genome. Nature Reviews Genetics.

[b35] Fleming MA, Potter JD, Ramirez CJ, Ostrander GK, Ostrander EA (2003). Understanding missense mutations in the BRCA1 gene: an evolutionary approach. Proceedings of the National Academy of Sciences of the United States of America.

[b36] Frazer KA, Ballinger DG, Cox DR, Hinds DA, Stuve LL, Gibbs RA, Belmont JW (2007). A second generation human haplotype map of over 3.1 million SNPs. Nature.

[b37] Fu YX, Li WH (1993). Statistical tests of neutrality of mutations. Genetics.

[b38] Gann PH, Hennekens CH, Ma J, Longcope C, Stampfer MJ (1996). Prospective study of sex hormone levels and risk of prostate cancer. Journal of the National Cancer Institute.

[b28] 1000 Genomes Project Consortium (2010). A map of human genome variation from population-scale sequencing. Nature.

[b39] Gillam SJ, Jarman B, White P, Law R (1989). Ethnic differences in consultation rates in urban general practice. British Medical Journal.

[b40] Gilthorpe MS, Lay-Yee R, Wilson RC, Walters S, Griffiths RK, Bedi R (1998). Variations in hospitalization rates for asthma among black and minority ethnic communities. Respiratory Medicine.

[b41] Girirajan S, Campbell CD, Eichler EE (2011). Human copy number variation and complex genetic disease. Annual Review of Genetics.

[b42] Gold DR, Rotnitzky A, Damokosh AI, Ware JH, Speizer FE, Ferris BG, Dockery DW (1993). Race and gender differences in respiratory illness prevalence and their relationship to environmental exposures in children 7 to 14 years of age. American Review of Respiratory Disease.

[b43] Green RE, Krause J, Briggs AW, Maricic T, Stenzel U, Kircher M, Patterson N (2010). A draft sequence of the Neandertal genome. Science.

[b44] Grochola LF, Vazquez A, Bond EE, Wurl P, Taubert H, Muller TH, Levine AJ (2009). Recent natural selection identifies a genetic variant in a regulatory subunit of protein phosphatase 2A that associates with altered cancer risk and survival. Clinical Cancer Reseach.

[b45] Hall JM, Lee MK, Newman B, Morrow JE, Anderson LA, Huey B, King MC (1990). Linkage of early-onset familial breast cancer to chromosome 17q21. Science 250.

[b46] Hamblin MT, Di Rienzo A (2000). Detection of the signature of natural selection in humans: evidence from the Duffy blood group locus. American Journal of Human Genetics.

[b47] Hamblin MT, Thompson EE, Di Rienzo A (2002). Complex signatures of natural selection at the Duffy blood group locus. American Journal of Human Genetics.

[b48] Han J, Kraft P, Nan H, Guo Q, Chen C, Qureshi A, Hankinson SE (2008). A genome-wide association study identifies novel alleles associated with hair color and skin pigmentation. PLoS Genetics.

[b49] Harpending HC, Pagel M (2002). Race: population genetics perspectives. Encyclopedia of Evolution.

[b50] Hurst LD (2009). Fundamental concepts in genetics: genetics and the understanding of selection. Nature Reviews Genetics.

[b51] Huttley GA, Easteal S, Southey MC, Tesoriero A, Giles GG, McCredie MR, Hopper JL (2000). Adaptive evolution of the tumour suppressor BRCA1 in humans and chimpanzees. Australian Breast Cancer Family Study. Nature Genetics.

[b25] International HapMap Consortium (2005). A haplotype map of the human genome. Nature.

[b52] Iskow RC, Gokcumen O, Lee C (2012). Exploring the role of copy number variants in human adaptation. Trends in Genetics.

[b53] Itan Y, Jones BL, Ingram CJ, Swallow DM, Thomas MG (2010). A worldwide correlation of lactase persistence phenotype and genotypes. BMC Evolutionary Biology.

[b54] Izagirre N, Garcia I, Junquera C, Alonso C, de la Rua S (2006). A scan for signatures of positive selection in candidate loci for skin pigmentation in humans. Molecular Biology and Evolution.

[b55] Jablonski NG, Chaplin G (2000). The evolution of human skin coloration. Journal of Human Evolution.

[b56] Jablonski NG, Chaplin G (2012). Human skin pigmentation, migration and disease susceptibility. Philosophical transactions of the Royal Society of London. Series B, Biological sciences.

[b57] Joffe B, Zimmet P (1998). The thrifty genotype in type 2 diabetes: an unfinished symphony moving to its finale?. Endocrine.

[b58] Kollias N, Sayre RM, Zeise L, Chedekel MR (1991). Photoprotection by melanin. Journal of photochemistry and photobiology. B, Biology.

[b59] Kreitman M (2000). Methods to detect selection in populations with applications to the human. Annual Reviews of Genomics and Human Genetics.

[b60] Kwiatkowski DP (2005). How malaria has affected the human genome and what human genetics can teach us about malaria. American Journal of Human Genetics.

[b61] Lamason RL, Mohideen MA, Mest JR, Wong AC, Norton HL, Aros MC, Jurynec MJ (2005). SLC24A5, a putative cation exchanger, affects pigmentation in zebrafish and humans. Science.

[b62] Lander ES, Linton LM, Birren B, Nusbaum C, Zody MC, Baldwin J, Devon K (2001). Initial sequencing and analysis of the human genome. Nature.

[b63] Laval G, Patin E, Barreiro LB, Quintana-Murci L (2010). Formulating a historical and demographic model of recent human evolution based on resequencing data from noncoding regions. PLoS ONE.

[b64] Le Souef PN, Goldblatt J, Lynch NR (2000). Evolutionary adaptation of inflammatory immune responses in human beings. Lancet.

[b65] Lee WH, Boyer TG (2001). BRCA1 and BRCA2 in breast cancer. Lancet.

[b66] Leroi AM, Koufopanou V, Burt A (2003). Cancer selection. Nature Reviews Cancer.

[b67] Lewin R (1987). Africa: cradle of modern humans. Science.

[b68] Lewontin RC, Krakauer J (1973). Distribution of gene frequency as a test of the theory of the selective neutrality of polymorphisms. Genetics.

[b69] Livingstone FB (1984). The Duffy blood groups, vivax malaria, and malaria selection in human populations: a review. Human Biology.

[b70] Louicharoen C, Patin E, Paul R, Nuchprayoon I, Witoonpanich B, Peerapittayamongkol C, Casademont I (2009). Positively selected G6PD-Mahidol mutation reduces Plasmodium vivax density in Southeast Asians. Science.

[b71] Macaulay V, Hill C, Achilli A, Rengo C, Clarke D, Meehan W, Blackburn J (2005). Single, rapid coastal settlement of Asia revealed by analysis of complete mitochondrial genomes. Science.

[b72] McDonald JH, Kreitman M (1991). Adaptive protein evolution at the Adh locus in Drosophila. Nature.

[b73] McGowan KA, Li JZ, Park CY, Beaudry V, Tabor HK, Sabnis AJ, Zhang W (2008). Ribosomal mutations cause p53-mediated dark skin and pleiotropic effects. Nature Genetics.

[b74] Mellars P (2006). A new radiocarbon revolution and the dispersal of modern humans in Eurasia. Nature.

[b75] Miller JE (2000). The effects of race/ethnicity and income on early childhood asthma prevalence and health care use. American Journal of Public Health.

[b76] Modiano D, Chiucchiuini A, Petrarca V, Sirima BS, Luoni G, Roggero MA, Corradin G (1999). Interethnic differences in the humoral response to non-repetitive regions of the Plasmodium falciparum circumsporozoite protein. American Journal of Tropical Medicine and Hygiene.

[b77] Myles S, Somel M, Tang K, Kelso J, Stoneking M (2007). Identifying genes underlying skin pigmentation differences among human populations. Human Genetics.

[b78] Nagy JD, Victor EM, Cropper JH (2007). Why don't all whales have cancer? A novel hypothesis resolving Peto's paradox. Integrative and comparative biology.

[b79] Neel JV (1962). Diabetes mellitus: a ‘thrifty’ genotype rendered detrimental by ‘progress’?. American Journal of Human Genetics.

[b80] Nielsen R, Bustamante C, Clark AG, Glanowski S, Sackton TB, Hubisz MJ, Fledel-Alon A (2005). A scan for positively selected genes in the genomes of humans and chimpanzees. PLoS Biology.

[b81] Nielsen R, Hellmann I, Hubisz M, Bustamante C, Clark AG (2007). Recent and ongoing selection in the human genome. Nature Reviews Genetics.

[b82] Norton HL, Kittles RA, Parra E, McKeigue P, Mao X, Cheng K, Canfield VA (2007). Genetic evidence for the convergent evolution of light skin in Europeans and East Asians. Molecular Biology and Evolution.

[b83] Novembre J, Di Rienzo A (2009). Spatial patterns of variation due to natural selection in humans. Nature Reviews Genetics.

[b84] Paiss T, Worner S, Kurtz F, Haeussler J, Hautmann RE, Gschwend JE, Herkommer K (2003). Linkage of aggressive prostate cancer to chromosome 7q31-33 in German prostate cancer families. European Journal of Human Genetics.

[b85] Patin E, Quintana-Murci L (2008). Demeter's legacy: rapid changes to our genome imposed by diet. Trends in Ecology & Evolution.

[b86] Pavard S, Metcalf CJ (2007). Negative selection on BRCA1 susceptibility alleles sheds light on the population genetics of late-onset diseases and aging theory. PLoS ONE.

[b87] Pavlicek A, Noskov VN, Kouprina N, Barrett JC, Jurka J, Larionov V (2004). Evolution of the tumor suppressor BRCA1 locus in primates: implications for cancer predisposition. Human Molecular Genetic.

[b88] Perry GH, Dominy NJ, Claw KG, Lee AS, Fiegler H, Redon R, Werner J (2007). Diet and the evolution of human amylase gene copy number variation. Nature Genetics.

[b89] Peto R, Roe FJ, Lee PN, Levy L, Clack J (1975). Cancer and ageing in mice and men. British Journal of Cancer.

[b90] Pickrell JK, Coop G, Novembre J, Kudaravalli S, Li JZ, Absher D, Srinivasan BS (2009). Signals of recent positive selection in a worldwide sample of human populations. Genome Research.

[b91] Prugnolle F, Manica A, Charpentier M, Guegan JF, Guernier V, Balloux F (2005). Pathogen-driven selection and worldwide HLA class I diversity. Current Biology.

[b92] Ptak SE, Przeworski M (2002). Evidence for population growth in humans is confounded by fine-scale population structure. Trends in Genetics.

[b93] Quintana-Murci L, Semino O, Bandelt HJ, Passarino G, McElreavey K, Santachiara-Benerecetti AS (1999). Genetic evidence of an early exit of Homo sapiens sapiens from Africa through eastern Africa. Nature Genetics.

[b94] Quintana-Murci L, Alcais A, Abel L, Casanova JL (2007). Immunology in natura: clinical, epidemiological and evolutionary genetics of infectious diseases. Nature Immunology.

[b95] Reich D, Green RE, Kircher M, Krause J, Patterson N, Durand EY, Viola B (2010). Genetic history of an archaic hominin group from Denisova Cave in Siberia. Nature.

[b96] Ribera JM, Oriol A (2009). Acute lymphoblastic leukemia in adolescents and young adults. Hematology/Oncology Clinics of North America.

[b97] Sabeti PC, Reich DE, Higgins JM, Levine HZ, Richter DJ, Schaffner SF, Gabriel SB (2002). Detecting recent positive selection in the human genome from haplotype structure. Nature.

[b98] Sabeti PC, Schaffner SF, Fry B, Lohmueller J, Varilly P, Shamovsky O, Palma A (2006). Positive natural selection in the human lineage. Science.

[b99] Sabeti PC, Varilly P, Fry B, Lohmueller J, Hostetter E, Cotsapas C, Xie X (2007). Genome-wide detection and characterization of positive selection in human populations. Nature.

[b100] Salem AH, Badr FM, Gaballah MF, Paabo S (1996). The genetics of traditional living: Y-chromosomal and mitochondrial lineages in the Sinai Peninsula. American Journal of Human Genetics.

[b101] Saunders MA, Hammer MF, Nachman MW (2002). Nucleotide variability at G6pd and the signature of malarial selection in humans. Genetics.

[b102] Sawyer SA, Hartl DL (1992). Population genetics of polymorphism and divergence. Genetics.

[b103] Schaffner SF, Foo C, Gabriel S, Reich D, Daly MJ, Altshuler D (2005). Calibrating a coalescent simulation of human genome sequence variation. Genome Research.

[b104] Seielstad MT, Minch E, Cavalli-Sforza LL (1998). Genetic evidence for a higher female migration rate in humans. Nature Genetics.

[b105] Shriver MD, Parra EJ, Dios S, Bonilla C, Norton H, Jovel C, Pfaff C (2003). Skin pigmentation, biogeographical ancestry and admixture mapping. Human Genetics.

[b106] Sironi M, Clerici M (2010). The hygiene hypothesis: an evolutionary perspective. Microbes and infection/Institut Pasteur.

[b107] Soejima M, Tachida H, Ishida T, Sano A, Koda Y (2006). Evidence for recent positive selection at the human AIM1 locus in a European population. Molecular Biology and Evolution.

[b108] Stajich JE, Hahn MW (2005). Disentangling the effects of demography and selection in human history. Molecular Biology and Evolution.

[b109] Stearns SC, Nesse RM, Govindaraju DR, Ellison PT (2010). Evolution in health and medicine Sackler colloquium: evolutionary perspectives on health and medicine. Proceedings of the National Academy of Sciences of the United States of America.

[b110] Stokowski RP, Pant PV, Dadd T, Fereday A, Hinds DA, Jarman C, Filsell W (2007). A genomewide association study of skin pigmentation in a South Asian population. American Journal of Human Genetics.

[b111] Sulem P, Gudbjartsson DF, Stacey SN, Helgason A, Rafnar T, Magnusson KP, Manolescu A (2007). Genetic determinants of hair, eye and skin pigmentation in Europeans. Nature Genetics.

[b112] Sun C, Huo D, Southard C, Nemesure B, Hennis A, Cristina Leske M, Wu SY (2011). A signature of balancing selection in the region upstream to the human UGT2B4 gene and implications for breast cancer risk. Human Genetics.

[b113] Swallow DM (2003). Genetics of lactase persistence and lactose intolerance. Annual Reviews of Genetics.

[b114] Tajima F (1989). Statistical method for testing the neutral mutation hypothesis by DNA polymorphism. Genetics.

[b115] Tang K, Thornton KR, Stoneking M (2007). A new approach for using genome scans to detect recent positive selection in the human genome. PLoS Biology.

[b116] Thomas F, Elguero E, Brodeur J, Roche B, Missé D, Raymond M (2012). Malignancies and high birth weight in human: which cancers could result from antagonistic pleiotropy?. Journal of Evolutionary Medicine.

[b117] Tishkoff SA, Varkonyi R, Cahinhinan N, Abbes S, Argyropoulos G, Destro-Bisol G, Drousiotou A (2001). Haplotype diversity and linkage disequilibrium at human G6PD: recent origin of alleles that confer malarial resistance. Science.

[b118] Tishkoff SA, Reed FA, Ranciaro A, Voight BF, Babbitt CC, Silverman JS, Powell K (2007). Convergent adaptation of human lactase persistence in Africa and Europe. Nature Genetics.

[b119] Tournamille C, Colin Y, Cartron JP, Le Van Kim C (1995). Disruption of a GATA motif in the Duffy gene promoter abolishes erythroid gene expression in Duffy-negative individuals. Nature Genetics.

[b120] Venter JC, Adams MD, Myers EW, Li PW, Mural RJ, Sutton GG, Smith HO (2001). The sequence of the human genome. Science.

[b121] Voight BF, Adams AM, Frisse LA, Qian Y, Hudson RR, Di Rienzo A (2005). Interrogating multiple aspects of variation in a full resequencing data set to infer human population size changes. Proceedings of the National Academy of Sciences of the United States of America.

[b122] Voight BF, Kudaravalli S, Wen X, Pritchard JK (2006). A map of recent positive selection in the human genome. PLoS Biology.

[b123] Von Behren J, Kreutzer R, Smith D (1999). Asthma hospitalization trends in California, 1983-1996. Journal of Asthma.

[b124] Weir BS, Hill WG (2002). Estimating F-statistics. Annual Review of Genetics.

[b125] Wilder JA, Mobasher Z, Hammer MF (2004). Genetic evidence for unequal effective population sizes of human females and males. Molecular Biology and Evolution.

[b126] Williams GC (1957). Pleiotropy, Natural Selection, and the Evolution of Senescence. Evolution.

[b127] Wissenbach U, Niemeyer BA, Fixemer T, Schneidewind A, Trost C, Cavalie A, Reus K (2001). Expression of CaT-like, a novel calcium-selective channel, correlates with the malignancy of prostate cancer. Journal of Biology and Chemistry.

[b128] Wooding S, Kim UK, Bamshad MJ, Larsen J, Jorde LB, Drayna D (2004). Natural selection and molecular evolution in PTC, a bitter-taste receptor gene. American Journal of Human Genetics.

[b129] Wooding S, Bufe B, Grassi C, Howard MT, Stone AC, Vazquez M, Dunn DM (2006). Independent evolution of bitter-taste sensitivity in humans and chimpanzees. Nature.

[b130] Yang Z (1998). Likelihood ratio tests for detecting positive selection and application to primate lysozyme evolution. Molecular Biology and Evolution.

[b131] Yi X, Liang Y, Huerta-Sanchez E, Jin X, Cuo ZX, Pool JE, Xu X (2010). Sequencing of 50 human exomes reveals adaptation to high altitude. Science.

